# Insights into Microbiome and Metabolic Signatures of Children Undergoing Peanut Oral Immunotherapy

**DOI:** 10.3390/children9081192

**Published:** 2022-08-09

**Authors:** Andrea C. Blackman, Santosh Thapa, Alamelu Venkatachalam, Thomas D. Horvath, Jessica K. Runge, Sigmund J. Haidacher, Kathleen M. Hoch, Anthony M. Haag, Ruth Ann Luna, Aikaterini Anagnostou

**Affiliations:** 1 Department of Pediatrics, Section of Immunology, Allergy and Retrovirology, Texas Children’s Hospital, Houston, TX 77030, USA; 2 Section of Allergy, Immunology & Retrovirology, Baylor College of Medicine, Houston, TX 77030, USA; 3 Department of Pathology and Immunology, Baylor College of Medicine, Houston, TX 77030, USA; 4 Texas Children’s Microbiome Center, Department of Pathology, Texas Children’s Hospital, Houston, TX 77030, USA

**Keywords:** food allergy, children, short chain fatty acids, alpha diversity, beta diversity, peanut, desensitization, tolerance

## Abstract

Background: Peanut oral immunotherapy has emerged as a novel, active management approach for peanut-allergic sufferers, but limited data exist currently on the role of the microbiome in successful desensitization. Objective: We examined the oral and gut microbiome in a cohort of 17 children undergoing peanut oral immunotherapy with the aim to identify the microbiome signatures associated with successful desensitization. We also set out to characterize their fecal metabolic profiles after successful therapy. Methods: Participants gradually built up their daily dose from 2 mg (starting dose) to 300 mg (maintenance dose) within approximately 40 weeks. We collected a buccal and stool specimen from each subject at two different time points: at baseline and post-therapy (1 month after reaching maintenance). The oral (buccal) and gut (fecal) microbiome was characterized based on sequencing of 16S rRNA gene amplicons with Illumina MiSeq. Fecal short chain fatty acid levels were measured using liquid chromatography-tandem mass spectrometry. Results: We report increased alpha diversity of the oral microbiome post-therapy and have also identified a significant increase in the relative abundance of oral Actinobacteria, associated with the desensitized state. However, the baseline gut microbiome did not differ from the post-therapy. Additionally, fecal short chain fatty acids increased after therapy, but not significantly. Conclusion: Our research adds to the limited current knowledge on microbiome and metabolic signatures in pediatric patients completing oral immunotherapy. Post-therapy increased trends of fecal fatty acid levels support a role in modulating the allergic response and potentially exerting protective and anti-inflammatory effects alongside successful desensitization. A better understanding of the microbiome-related mechanisms underlying desensitization may allow development of smarter therapeutic approaches in the near future. Clinical implication: The oral microbiome composition is altered following successful peanut oral immunotherapy, with a significant increase in alpha diversity and the relative abundance of phylum Actinobacteria. Capsule summary: Significant microbiome changes in children completing peanut immunotherapy include increase in alpha-diversity and overrepresentation of Actinobacteria in the oral microbiome, and increased trends for fecal short chain fatty acids, suggesting a protective effect against the allergic response.

## 1. Introduction

Food allergies are common and their prevalence is increasing [1,2]. An estimated 2% of US children are reported to suffer from peanut allergy, with significant effect on their quality of life [3,4,5]. Peanut-allergic children frequently display anxiety, caused by their fear of accidental exposure with potentially severe reactions, and life-threatening events. There are other multiple daily challenges for these children, including dietary and social restrictions, in addition to psychological issues at school (such as bullying) [6,7,8,9]. Peanut allergy is a lifelong condition that rarely resolves, with only 1 in 5 children outgrowing it naturally [10] and there is currently no cure. In recent years, oral immunotherapy has emerged as a novel intervention and potentially disease-modifying therapy. Peanut oral immunotherapy (POIT) has so far shown promising results in successfully desensitizing the majority of allergic patients [11,12,13,14,15,16,17,18], resulting in the first FDA-approved drug- Palforzia (peanut (*Arachis hypogaea*) allergen powder-dnfp)- for peanut allergy therapy.

Identifying the root cause of food allergies has been the goal of research for many years. Emerging evidence suggests that the microbiome contributes to the development and manifestations of food allergy. In fact, dysregulation in the homeostatic interaction between the host and the microbiome (“gut dysbiosis”) is reported to precede the development of food allergy [19,20]. The microbiome also appears to affect food tolerance via the secretion of microbial metabolites such as short chain fatty acids (SCFAs); humans consume prebiotic fiber, which is metabolized by resident microbes in the gut to create SCFAs. These, in turn, regulate immune responses [21,22,23]. Limited data exist currently on the role of the microbiome in oral immunotherapy. The aim of our work was to identify taxa associated with desensitization and to characterize metabolic signatures after successful therapy.

## 2. Materials and Methods

### 2.1. Patient Enrollment and Ethical Approval

We enrolled seventeen children 4–15 years old with a physician-diagnosed peanut allergy undergoing POIT treatment as previously described [24]. Written informed consent was obtained from the parents of all participants, in addition to age-appropriate patient assent for both POIT and microbiome studies. Children with uncontrolled severe asthma, uncontrolled atopic dermatitis, severe allergic rhinitis, a diagnosis of eosinophilic esophagitis or any other chronic gastrointestinal disorder were excluded. Information regarding age, gender, mode of delivery, infant feeding (breastfeeding or formula), antibiotic use, household pets, and probiotic use were collected for all participants. Participants gradually built up their peanut OIT daily dose from 2 mg (starting dose) to 300 mg (maintenance dose) within approximately 40 weeks. This research was approved by the Institutional Review Board at Baylor College of Medicine.

### 2.2. Sample Collection

Buccal and stool specimens were collected from each subject at two different time points: pre-POIT (baseline) and post-POIT (1 month after reaching maintenance).

#### 2.2.1. Buccal Samples

We collected two buccal swabs from each of the 17 participants, one pre-POIT and another post-POIT, as described above. Samples were collected by inserting the sterile Hydraflock swab with break point (Puritan, Guilford, ME, USA) on one side of the mouth between the inside of the cheek and the upper gum. The swab was pressed firmly and twirled against the inside of the inner cheek for 30 s to collect cells, using an up and down motion from front to back and back to front. After collection, the swab was placed in a 750-µL volume of PowerBead Solution (Qiagen, Hilden, Germany), labeled, and sent to the Texas Children’s Microbiome Center (TCMC) for microbiome characterization. Buccal samples were stored at −80 °C until further processing.

#### 2.2.2. Stool Samples

A total of 34 stool samples were collected aseptically from 17 participants (two samples per participant: one pre-POIT and another post-POIT) and were subsequently sent to the TCMC for gut microbiome characterization. Fecal aliquots were also used for liquid chromatography-tandem mass spectrometry (LC-MS/MS)-based targeted metabolomics analysis of specific short-chain fatty acids (SCFAs). Stool samples were stored at −80 °C until further processing.

### 2.3. DNA Extraction

The DNeasy PowerSoil Kit (Qiagen, Hilden, Germany) was used for extraction of microbial genomic DNA from both buccal and stool samples. For buccal swabs, we followed the manufacturer’s recommendation with minor modifications as mentioned below. Briefly, a 200-µL volume of PowerBead Solution in PowerBead Tube was removed and replaced with a 300-µL of buccal swab-suspended PowerBead Solution prior to extraction. DNA extraction from stool samples was performed using the QIAcube (Qiagen, Hilden, Germany) automated platform at default settings, except the 60-µL elution volume. The resultant DNA was quantified on the Qubit^®^ 2.0 fluorometer (Thermo Fisher Scientific, Inc., Wilmington, DE, USA) using the Invitrogen™ Qubit™ dsDNA HS Assay Kit. The DNA samples were stored at −80 °C until processing.

### 2.4. 16S rRNA Sequencing

The V4 region of the 16S rRNA gene was amplified by PCR as previously described [25]. The pooled amplicon libraries were sequenced using a 500 cycle v2 chemistry kit on an Illumina MiSeq platform. Negative controls (no template PCR controls, *n* = 4 and extraction controls, *n* = 4) were sequenced in parallel with the samples (*n* = 68) to monitor potential background noise.

### 2.5. Bioinformatic Analysis of 16S Data

The raw reads were processed as previously described [26]. The sequences were then quality filtered using the LotuS pipeline (v1.462) [27] and processed as previously described [26,28]. The unclassified sequences at the kingdom level and non-bacterial sequences including mitochondria, chloroplast, cyanobacteria, and archaea were removed from the biom file before proceeding with analysis. The decontam R package [29] was used to identify and remove possible contaminant OTUs from the dataset. Only samples with >1000 reads post-decontam were included in the downstream analysis.

The microbial community diversity (alpha and beta) and relative abundance of bacterial taxa in each sample was calculated using the phyloseq R-package (v1.32.0). Diversity analyses were performed in a dataset generated after evenly subsampling (without replacement) the data to the lowest sequencing depth (1467 reads for buccal swabs and 2295 reads for stool samples) to minimize the potential bias of unequal sequencing depth among samples. Observed OTUs, Pielou’s evenness and Shannon index were employed to estimate alpha diversity. Principal coordinate analysis (PCoA) plots of Jaccard index (presence or absence), Bray-Curtis dissimilarity index (abundance-weighted) and both unweighted and weighted UniFrac distance metrics [30] were used to assess the beta diversity between samples.

### 2.6. Fecal SCFAs Measurement

The fecal SCFA content was measured by using a liquid chromatography-mass spectrometry (LC-MS) system located in the Metabolomics and Proteomics Laboratory of the Texas Children’s Microbiome Center. All stool samples were homogenized with 1.25 mL of 50% acetonitrile, refrigerated overnight at 4 °C, and centrifuged for 10 min at 18,000 rcf. A 500-µL aliquot volume of supernatant was provided for each sample. The LC-MS/MS system used for this study included a Shimadzu (Kyoto, Japan) Nexera-XR ultra-high performance liquid chromatography (UHPLC) system coupled to a Sciex (Framingham, MA, USA) 6500 QTRAP hybrid triple-quadrupole/linear ion trap mass spectrometer. Instrument control and data acquisition were performed using Sciex Analyst^®^ (version 1.6.2). Peak integration, weighted (1/x) linear regression analysis of calibration curves, and unknown sample analysis was performed using Sciex MultiQuant™ 3.0.1 (version 3.0.6256.0).

Prior to quantitative analysis by LC-MS/MS, the SCFAs (acetic acid, propionic acid, butyric acid, isobutyric acid, pentanoic acid, isopentanoic acid, and 2-methybutyric acid) content of the fecal samples was derivatized as previously described [31]. Chromatographic separation of the SCFA-analide derivatives was performed using a reversed-phase chromatographic system consisting of a 5-micron Viva BiPh Biphenyl (300 Å, 100 × 1 mm) analytical column coupled to a 5-micron Viva Biphenyl (10 × 2.1 mm) guard cartridge from Restek (Bellefonte, PA, USA). The MS system was operated in positive ion mode with the TurboIonSpray^®^ electrospray ionization (ESI) probe installed in the TurboV™ ion source. Details on the sample/reagents preparation, mobile phase system, source voltages, gas settings, and molecule specific parameters are included in Text S1 in the Online Repository.

### 2.7. Statistical Analysis

Statistical analyses were performed using R-software (v4.0.0) [32]. Alpha diversity between pre-POIT and post-POIT samples was compared using the Wilcoxon rank sum test. Pearson’s correlation analysis was performed to test the association between bacterial (alpha) diversity and age of the participants. Permutational multivariate analysis of variance (PERMANOVA) was used to compare the beta diversity between pre-POIT and post-POIT samples using the vegan R-package (v2.5.6) [33]. PERMANOVA was also used on beta diversity metrics to test the effect of clinical and demographic variables on the microbiome composition. A significant PERMANOVA result was further tested if the difference exists in the location or dispersion of the samples using a permutation-based test of homogeneity of dispersion (PERMDISP). The Wilcoxon rank sum test was also used to compare the fecal SCFAs concentrations pre- and post-POIT. Differences in the microbial signatures pre- and post-POIT at phylum and genus levels were compared using the Wilcoxon rank sum test. For this analysis, we selected the bacterial phyla and genera with >1% relative abundance in at least one sample to minimize the bias of many very low abundant taxa on multiple testing. The Benjamini–Hochberg (BH) correction was applied to control the false-discovery rate (FDR) in multiple hypotheses testing. *p*-values <0.05 were considered significant.

## 3. Results

### 3.1. Characteristics of the Study Cohort

The study cohort included 17 peanut-allergic children, 4–15 years (median = 7 years) (Table 1 and Table S1). Atopic dermatitis (76%) was the most common reported co-morbidity, followed by allergic rhinitis (71%), and asthma (41%). Multiple food allergies were reported in 71% children. Forty-one percent of participants had a sibling with food allergy. More than half of the participants had pets in their household. A total of 71% were reportedly breastfed. Five participants (29%) received antibiotics and three (18%) received probiotics while undergoing POIT. The demographics and clinical characteristics of individual subjects are shown in Table S2.

### 3.2. Microbiome Sequencing Results

**Figure 1:**
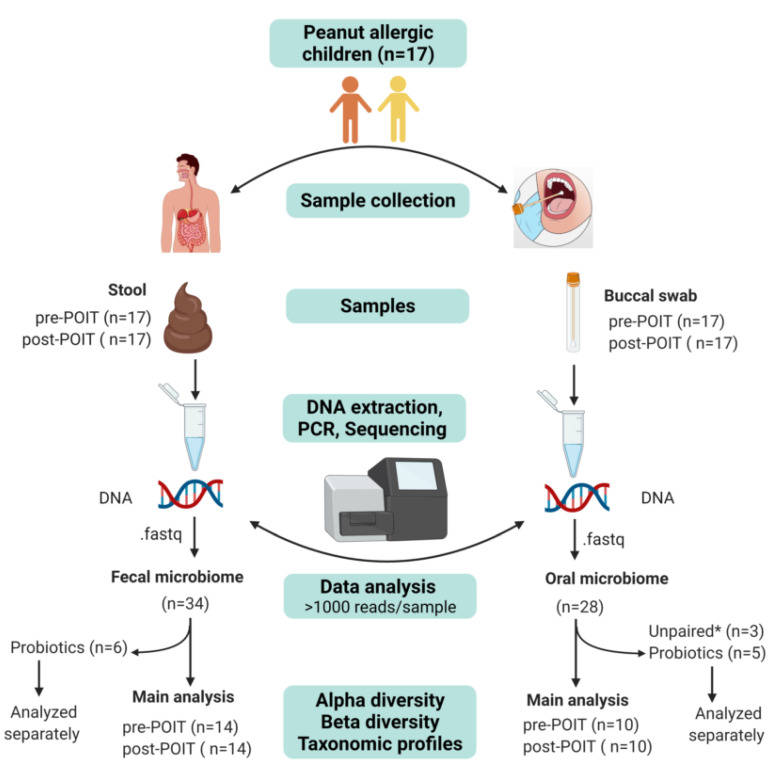
Flow chart for sample collection and data analysis. * Three unpaired (i.e., without both pre-POIT and post-POIT) buccal samples were excluded from the analysis.

Sixty-eight MiSeq libraries generated from the buccal (*n* = 34) and stool (*n* = 34) samples produced a total of 1,796,806 raw reads. After removing the non-bacterial reads and contaminant OTUs identified by decontam (see Figures S1 and S2 in the Online Repository), 6 buccal samples were excluded because of <1000 quality-filtered reads/sample. All stool samples had >1000 quality-filtered reads/sample. From the 62 samples (28 buccal and 34 stool), a total of 1,783,697 (buccal = 1,121,482 and stool = 662,215) quality-filtered reads (average per sample: buccal  =  40,053; stool = 19,477) were generated. Eleven samples (5 buccal and 6 stool) collected from three subjects taking probiotics were analyzed separately. Finally, three unpaired buccal samples (i.e., without both pre-POIT and post-POIT samples) were excluded from the analysis, leaving a total of 20 matched buccal samples (pre-POIT = 10 and post-POIT = 10) and 28 matched stool samples (pre-POIT = 14 and post-POIT = 14) for the main analysis. The overall sequencing and data analysis workflow with the number of samples are illustrated in Figure 1. Rarefaction curves of the observed OTUs plotted against the sequencing read depths (Figure S3) indicate that the samples had adequate depth for further analysis.

### 3.3. Effects of Demographics and Allergic Co-Morbidities on the Microbiome

The age of participants did not correlate with alpha diversity of either oral (buccal) or gut (fecal) microbiome (Figure S4). Additionally, the mode of delivery, infant feeding pattern, household pets, allergic co-morbidities, and use of antibiotics during POIT had no effect on the oral or gut microbiome composition (beta diversity). However, the oral (but not gut) microbial composition was significantly (PERMANOVA *p* < 0.05) different after successful POIT treatment (Table S3).

### 3.4. Oral Microbiome before and after POIT

**Figure 2:**
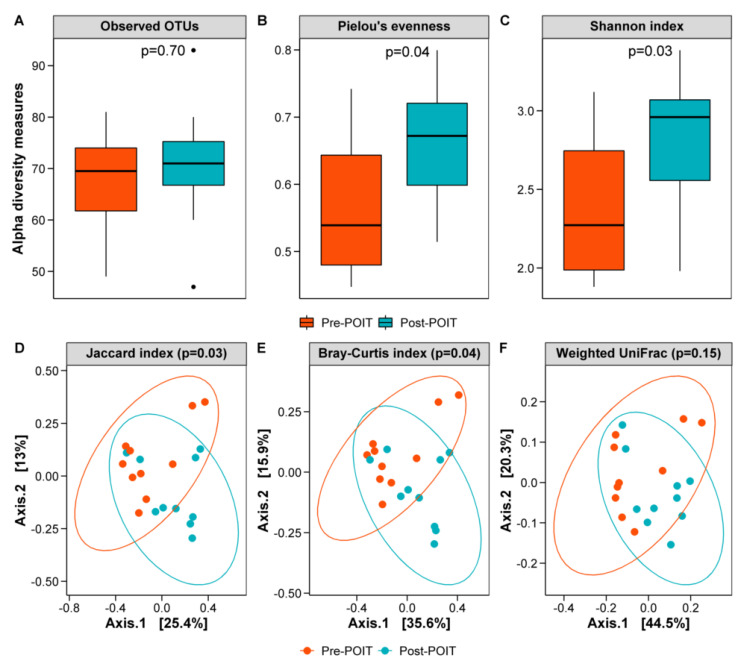
Oral bacterial diversity pre- and post-POIT. Number of observed OTUs (**A**), Pielou’s evenness (**B**) and Shannon index (**C**) were increased in the post-POIT group than the baseline (pre-POIT). Wilcoxon rank sum test *p* < 0.05 was considered statistically significant. PCoA plots of the Jaccard (**D**) and Bray–Curtis (**E**) indices showed differences in bacterial composition (beta diversity) between the pre- and post-POIT groups (PERMANOVA *p* < 0.05), but not with the UniFrac distance (**F**). The first two coordinates that explained the largest fraction of variably in the data was plotted in the PCoA plots.

While there was a trend toward an increase in richness (observed OTUs, Figure 2A) of the oral (buccal) microbiome post-POIT (Wilcoxon *p* = 0.70), both Pielou’s evenness (Figure 2B) and Shannon diversity index (Figure 2C) were significantly increased post-POIT compared to pre-POIT (Pielou’s: *p* = 0.04, Shannon: *p* =  0.03). With respect to beta diversity, the Jaccard (Figure 2D) and Bray–Curtis dissimilarity (Figure 2E) indices demonstrated compositional differences in the oral microbiome between pre- and post-POIT (PERMANOVA *p* < 0.05, PERMDISP *p* > 0.05). However, UniFrac (phylogenetic) distance-based beta diversity ordinations did not reveal significant differences in the oral microbiome before and after POIT (see Figure 2F for weighted UniFrac and Figure S5 for unweighted UniFrac results).

**Figure 3:**
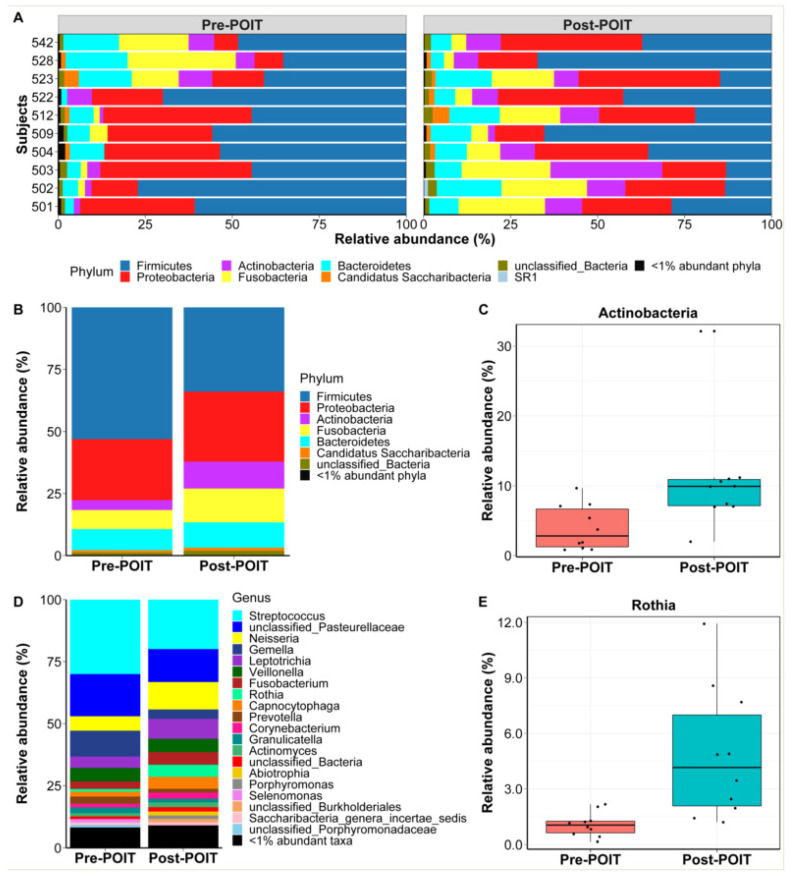
Oral bacterial composition pre- and post-POIT. Phylum level oral microbiome composition in individual participants before (pre) and after (post) POIT treatment (**A**). Phylum level taxa summary (mean relative abundance) of the oral bacteria (**B**) and differently abundant oral Actinobacteria phylum (**C**) between the groups. Genus level summary of bacterial taxa in the oral microbiome (**D**) and differently abundant genus *Rothia* (**E**) between the groups.

Although individual oral microbiome signatures varied between the participants (Figure 3A), 80% (8/10) of them had increased abundance of Actinobacteria phylum, and decreased abundance of Firmicutes after POIT treatment. Overall, the phylum level data (Figure 3B) demonstrated that the oral microbiome post-POIT had significantly increased abundance of Actinobacteria (Figure 3C) compared to pre-POIT (*p*  <  0.05, FDR-corrected). At the genus level (Figure 3D), there was increased abundance of *Rothia* (belonging to the phylum Actinobacteria) post-POIT (Figure 3E) in the oral microbiome compared with pre-POIT (*p*  <  0.05, FDR-corrected).

### 3.5. Gut Microbiome before and after POIT

There was no change in the gut (fecal) alpha diversity between pre- and post-POIT groups (Figure S6). Beta diversity of the gut microbiome also did not show any significant difference between pre- and post-POIT samples (Figure S7).

**Figure 4:**
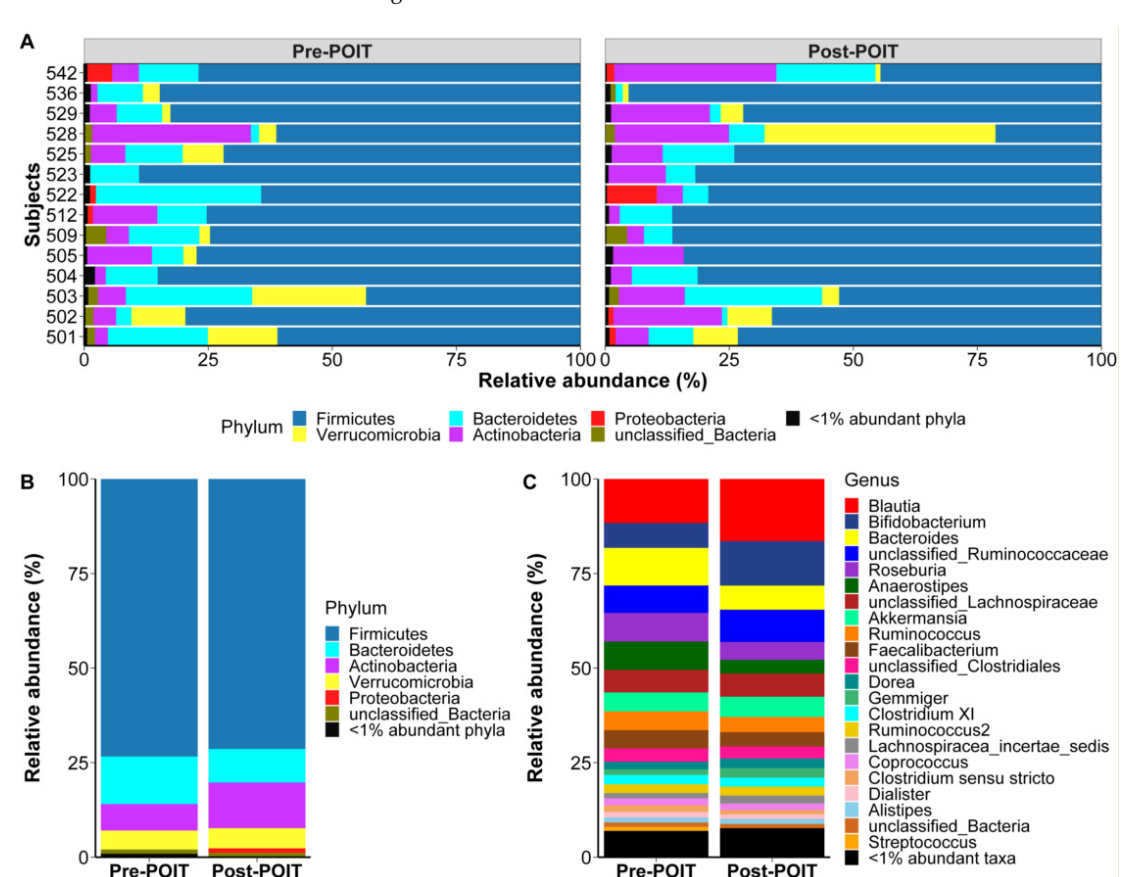
Fecal bacterial composition pre- and post-POIT. Phylum level fecal microbiome composition in individual participants before (pre) and after (post) POIT treatment (**A**). Phylum (**B**) and genus (**C**) levels taxa summary (mean relative abundance) of the fecal bacteria did not differ between the pre-POIT and post-POIT groups.

Individual gut microbiome signatures showed variation between participants (Figure 4A). However, the mean relative abundance (%) of bacterial taxa at the phylum (Figure 4B) and genus (Figure 4C) levels was not significantly different between pre- and post-POIT groups. The major phyla [mean relative abundance (%)] in pre-POIT and post-POIT groups were Firmicutes (pre: 73, post: 71), Bacteroidetes (pre: 13, post: 9), and Actinobacteria (pre: 7, post: 12). Although the genera [mean relative abundance (%)] *Blautia* (pre: 12, post: 16) and *Bifidobacterium* (pre: 7, post: 12) were higher, and *Bacteroides* (pre: 10, post: 6), *Roseburia* (pre: 8, post: 5), *Anaerostipes* (pre: 7, post: 4), and *Faecalibacterium* (pre: 5, post: 4) were lower in post-POIT group compared with pre-POIT, these values did not achieve statistical significance.

### 3.6. Fecal SCFAs Profiles in Pre-POIT and Post-POIT Samples

**Figure 5:**
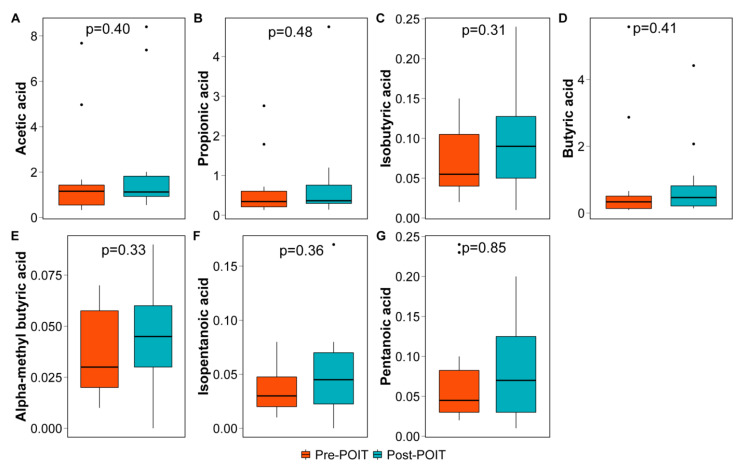
Fecal short chain fatty acids concentrations pre- and post-POIT. SCFAs level was measured as µmole/g wet feces. (**A**) acetic (**B**) propionic (**C**) isobutyric (**D**) butyric (**E**) alpha-methyl-butyric (**F**) isopentanoic (**G**) pentanoic acids.

We found a trend toward an increase in the fecal SCFAs concentrations in post-POIT group for acetic (Wilcoxon *p* = 0.40), propionic (*p* = 0.48), isobutyric (*p* = 0.31), butyric (*p* = 0.41), alpha-methyl-butyric (*p* = 0.33), isopentanoic (*p* = 0.36), and pentanoic (*p* = 0.85) acids, although these values did not reach statistical significance (Figure 5). At the individual level, some participants showed decrease in SCFAs levels after successful treatment while others had dramatic increase in their concentrations post-POIT (Figure S8).

### 3.7. Microbiome and SCFAs Levels in Patients Taking Probiotics

The oral microbiome composition before and after POIT was compared only in two participants as the pre-POIT sample from a third patient receiving probiotics failed to cross the 1000 reads cutoff. In both cases, oral abundance of genus *Streptococcus*, belonging to phylum Firmicutes, was decreased after POIT (Figure S9). Firmicutes and Bacteroidetes were the major gut phyla both pre- and post-POIT while *Bacteroides*, the most abundant genus, were generally decreased after therapy (Figure S10). Fecal SCFAs concentrations were varied by individuals (Table S4).

## 4. Discussion

To our knowledge this is the first study to describe microbiome and metabolic profiles in children successfully completing POIT. Results from our cohort of 17 peanut-allergic participants show a significant increase in alpha diversity of the oral microbiota after treatment. No significant difference was noted in the alpha diversity of the gut microbiota. This may be due to the fact that peanut is first encountered immunologically by the oral mucosa and has a more significant effect there. It has been noted previously that children developing allergic disease have lower diversity of salivary bacteria at 7 years of age [34]. This observation may indirectly suggest that a shift toward a non-reactive profile is associated with increased oral alpha diversity.

Increased microbial gut diversity has been suggested to reflect an immunomodulatory effect of OIT on the host immune system in successful desensitization [35]. A recent publication that retrospectively investigated stool samples collected from seven adult participants who received POIT, reported a significant increase in alpha diversity after treatment, but no change in beta diversity [35]. Similarly, in our cohort, beta diversity did not show any significant difference in either oral or gut microbiome between baseline and end of treatment. Results from subjects who are unsuccessful in achieving desensitization would also be of interest for comparison purposes, but no such studies are currently available.

Low gut microbial diversity has been associated with risk of allergic disease (linked to both asthma and eczema development [36,37]) in research studies. In contrast, a trial of 141 infants from five different centers in the United States noted that differences in gut bacterial diversity could not predict resolution or persistence of egg allergy at age 8 years [38]. In a mouse model, where germ-free mice were colonized with feces from either healthy or cow’s milk allergic (CMA) infants, Feehly et al. also noted no difference in community diversity and evenness between the healthy and CMA colonized mouse groups [39]. This inconsistency in reported results may reflect the fact that there is not always a beneficial association between increased microbiome diversity and disease protection or tolerance development. Additionally, it is very likely that the role of the microbiome cannot be captured by a single factor (such as alpha or beta diversity), but involves more complex interactions between different taxa and their metabolic effects [38].

Gut dysbiosis likely precedes the development of food allergy [19,39,40,41] and multiple microbial orders have been implicated in this process [42,43,44]. Studies evaluating the gut microbiome have revealed unique microbial differences in patients with food allergies compared to healthy patients, but no specific taxa have been consistently associated with food allergies and no data exist currently for children completing POIT [19]. Food allergy is a complex and heterogenous disease and studies often involve diverse patient populations and different enrolment criteria, therefore some inconsistency in results is expected.

In our cohort, analysis of the oral microbiome at the phylum level indicated the enrichment of Actinobacteria; bacterial species from the above family have been shown to promote immune tolerance and to have protective effects in food allergy [45,46]. At the genus level, we noted an increased abundance of *Rothia* post POIT. *Rothia* is part of the normal oropharyngeal flora and therefore an oral commensal. Previous reports have suggested that commensal bacteria may protect against food allergen sensitization [40] whereas commensal dysbiosis promotes the development of food allergy [47]. It is likely therefore that an increased abundance of *Rothia* reflects a shift toward tolerance development in our cohort and exerts a protective effect against peanut exposure.

For the gut microbiome, no significant differences were seen at phylum level, but *Bifidobacterium* was noted to have an increased relative abundance post treatment in our cohort. *Bifidobacteria* and their metabolites have been shown to induce peripheral regulatory T (Treg) cells that control inflammation and promote tolerance in murine models and infant studies [45,46,48,49]. Another recent study reported overrepresentation of five Clostridial species, one Firmicutes bacterium, and one Bacteroides species in stool samples of adult subjects after POIT compared with baseline [35]. In contrast, Firmicutes and Bacteroidetes phyla were underrepresented in our pediatric cohort. Age of participants and variations in up dosing schedules may play a role in the differing results. Individual oral microbiome (buccal) and gut microbiome (stool) signatures varied significantly between our participants, reinforcing the notion that both individual and group variability is a common observation in microbiome studies.

Our work is the first to investigate changes in short chain fatty acids (SCFAs) levels within the context of POIT in human subjects. We noted an increase in measured SCFA levels post treatment compared to baseline for acetic, propionic, isobutyric, butyric, alpha-methyl-butyric, isopentanoic and pentanoic acid. Although the difference did not reach statistical significance, likely due to the small sample size, these results are in line with the reported protective and anti-inflammatory effects that have been attributed to high SCFA levels. SCFAs have direct immune-modulatory effects and play an essential role in regulating protective and inflammatory responses [50,51,52]. Butyrate supplementation is shown to increase activated FoxP3+ Treg cells and stimulate the production of protective cytokines (IFN-γ and IL-10) in peripheral blood mononuclear cells [22]. Interestingly, both Treg induction and protective cytokines have been associated with successful POIT [53].

Additionally, our findings are in line with emerging evidence on the SCFAs’ importance in tolerance development, especially for butyrate [52]. In studies with pediatric patients with CMA, the ingestion of hydrolyzed formula containing a butyrate-producing probiotic increased fecal butyrate levels and was associated with the acquisition of immune tolerance [54]. In a murine model of cow’s milk allergy it was demonstrated that butyrate supplementation enhances OIT desensitization [55]. Our results also reinforce reports from food allergy prevention studies, showing a protective effect in subjects with elevated SCFAs levels. For example, children with the highest levels of butyrate and propionate at the age of one year were less likely to be diagnosed as food-allergic at 3 and 6 years [56].

In summary, we found increased alpha diversity of the oral microbiome post-POIT and have also identified a significant increase in the relative abundance of oral Actinobacteria, associated with the desensitized state. Additionally, fecal SCFAs showed an increasing trend after successful peanut oral immunotherapy.

Limitations of our work include the lack of control group, the small number of patient samples studied, and the exclusion of a few samples due to low number of reads. Additionally, oral food challenges were not part of our protocol. Given the trends in microbial and metabolic shifts that we identified, a larger sample size with the inclusion of a placebo arm will be necessary to capture the full complexity of the differences in microbiome and metabolome between pre- and post-POIT.

Strengths of the study include the use of a well-characterized cohort of peanut-allergic children undergoing POIT, use of well-established protocols/tools in microbiome/metabolomics sciences, and expertise in microbiome/metabolomics studies. Additionally, the microbiome and metabolic profiles of peanut-allergic children after successful completion of POIT provided important information that has the potential to inform future therapeutic interventions.

Our findings provide the benchmark information on the oral and gut microbiome composition and metabolic changes in peanut-allergic children undergoing POIT. Future studies using whole genome-based shotgun sequencing, targeted/untargeted metabolomics, and proteomics may provide new information about the functional roles of the microbiome in oral immunotherapy and potentially leads to the development of microbiome-modulating therapies in combination with or independent of POIT.

## Figures and Tables

**Table 1:** Cohort characteristics and early life information.

Variable	Peanut Allergic Cohort (*n* = 17)
Demographics	
Age (years)	7 ± 3
History of atopy	
Atopic dermatitis	13 (76%)
Allergic rhinitis	12 (71%)
Asthma	8 (47%)
Multiple food allergies	12 (71%)
Mode of delivery	
Vaginal	5 (29%)
Cesarean-section	12 (71%)
Feeding pattern	
Breastfed	12 (71%)
Formula	5 (29%)
Medications	
Antibiotics	5 (29%)
Probiotics	3 (18%)
Birth place-Urban	15 (88%)
Pets	9 (53%)
Dog	7
Cats and dogs	1
Rabbit	1
History of food allergies in sibling(s)	7 (41%)

Values are presented as number (%) or median ± standard deviation.

## Data Availability

All data supporting the findings of this study are included in the main text of the manuscript and the supplemental files. The raw microbiome and metabolome data are available on request from the corresponding author. The raw data are not publicly available due to lack of prior consent from the participants.

## Supplementary Materials

The following are available online at https://www.mdpi.com/article/10.3390/children9081192/s1, Figure S1: Representative contaminant OTUs identified in buccal samples using decontam R-package. Figure S2: Contaminant OTUs identified in stool samples using decontam R-package. Figure S3: Rarefaction curves of observed OTUs vs. read depths. Figure S4: Correlations between alpha diversity and age of the participants. Figure S5: Unweighted UniFrac distance-based oral beta diversity in pre- and post-POIT. Figure S6: Alpha diversity of the fecal microbiome pre- and post-POIT. Figure S7: Beta diversity of the fecal microbiome pre- and post-POIT. Figure S8: Fecal short chain fatty acids levels in individual subjects pre- and post-POIT. Figure S9: Oral bacterial composition in participants taking probiotics during POIT. Figure S10: Fecal bacterial composition in participants taking probiotics during POIT. Table S1: Pre POIT peanut specific IgE and Arah 2 levels and POIT related-symptoms during each up-dosing level. Table S2: Cohort characteristics and demographics of individual participants. Table S3: Effects of various factors on the oral and gut microbiome composition, as measured by PERMANOVA test of Bray-Curtis dissimilarity index. Table S4: Fecal SCFAs concentrations in participants taking probiotics during POIT. Table S5: Molar concentration and the volume dilutions to prepare 100 mM stock solutions for each SCFA compound measured. Table S6: Serial dilution scheme for the N-phenyl-SCFA derivatized Calibration Standards using the WIS-B solution as the diluent. Table S7: Mass spectrometric pa-rameters for each of the N-Phenyl-SCFA derivative and N-[13C6]-Phenyl-SCFA derivatives. Ab-breviations include mass-filtering quadrupole (Q1 and Q3), collision energy (CE), declustering po-tential (DP), entrance potential (EP), and cell-exit potential (CXP). Text S1: Detailed protocol for the measurement of fecal short chain fatty acids. Reference [57] is cited in the supplementary materials.

## Abbreviations

IFN-γ	Interferon gamma
IL-10	Interleukin-10
POIT	peanut oral immunotherapy
SCFAs	short chain fatty acids
LC-MS/MS	liquid chromatography-tandem mass spectrometry
Treg	T regulatory
