# Supplementary material for: Insights into Microbiome and Metabolic Signatures of Children Undergoing Peanut Oral Immunotherapy
Source: Children (Basel). 2022 Aug 9;9(8):1192. doi: 10.3390/children9081192 (PMC9406383; doi:10.3390/children9081192)
Supplement: Supplementary file 1 [file children-09-01192-s001.zip › children-1801420-supplementary.pdf]

Supplementary Materials

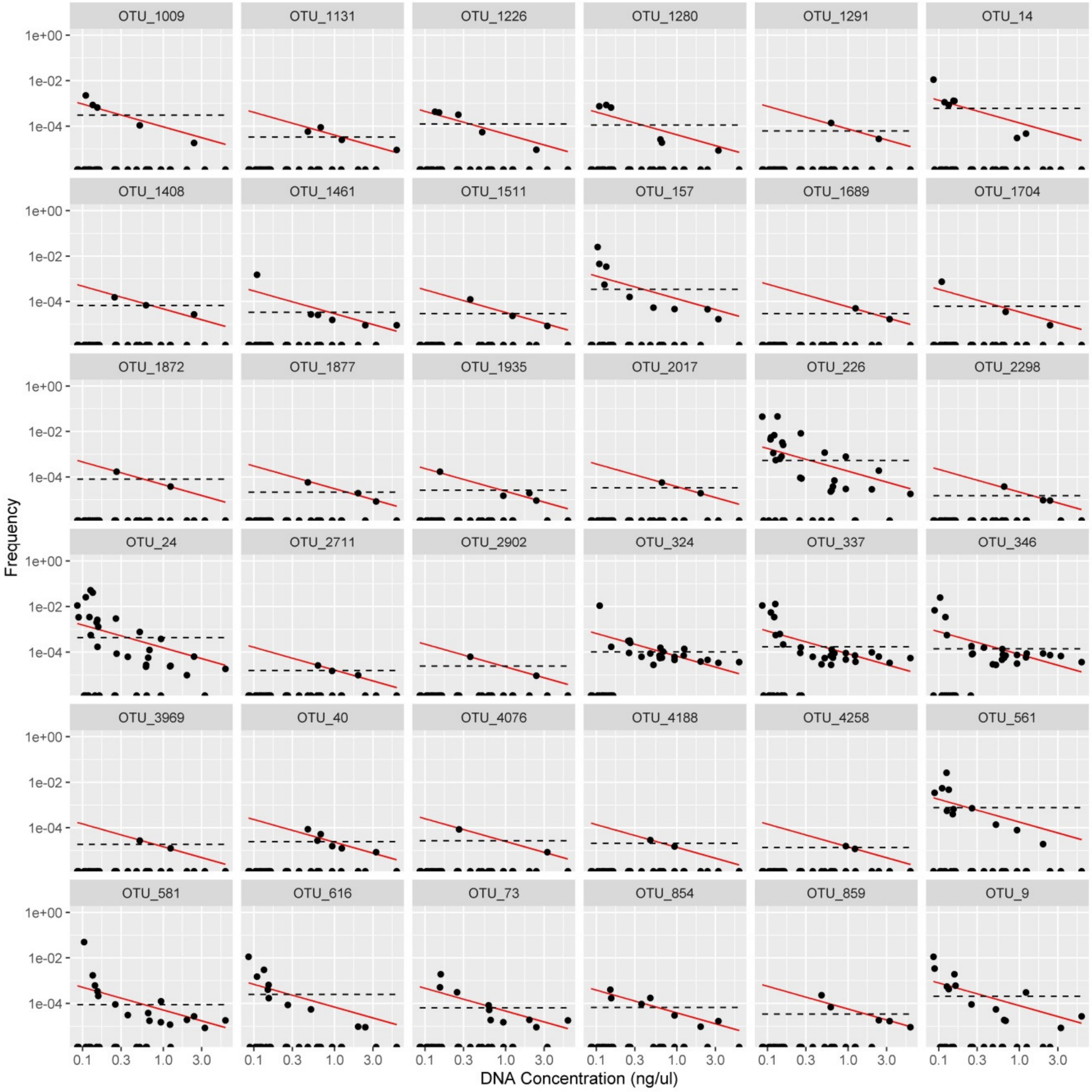

Figure S1. Representative contaminant OTUs identified in buccal samples using decontam R-package. The frequencies of OTUs were inspected as a function of DNA concentration measured by Qubit assay prior to MiSeq sequencing. An OTU is classified as contaminant or non-contaminant by comparing its associated score statistic P (decontam score) to the default classification threshold of 0.1. The frequency of all contaminant OTUs showed negative

correlation with the total DNA concentration (red line). The horizontal black dot line represents the expected frequency of non-contaminant OTUs.

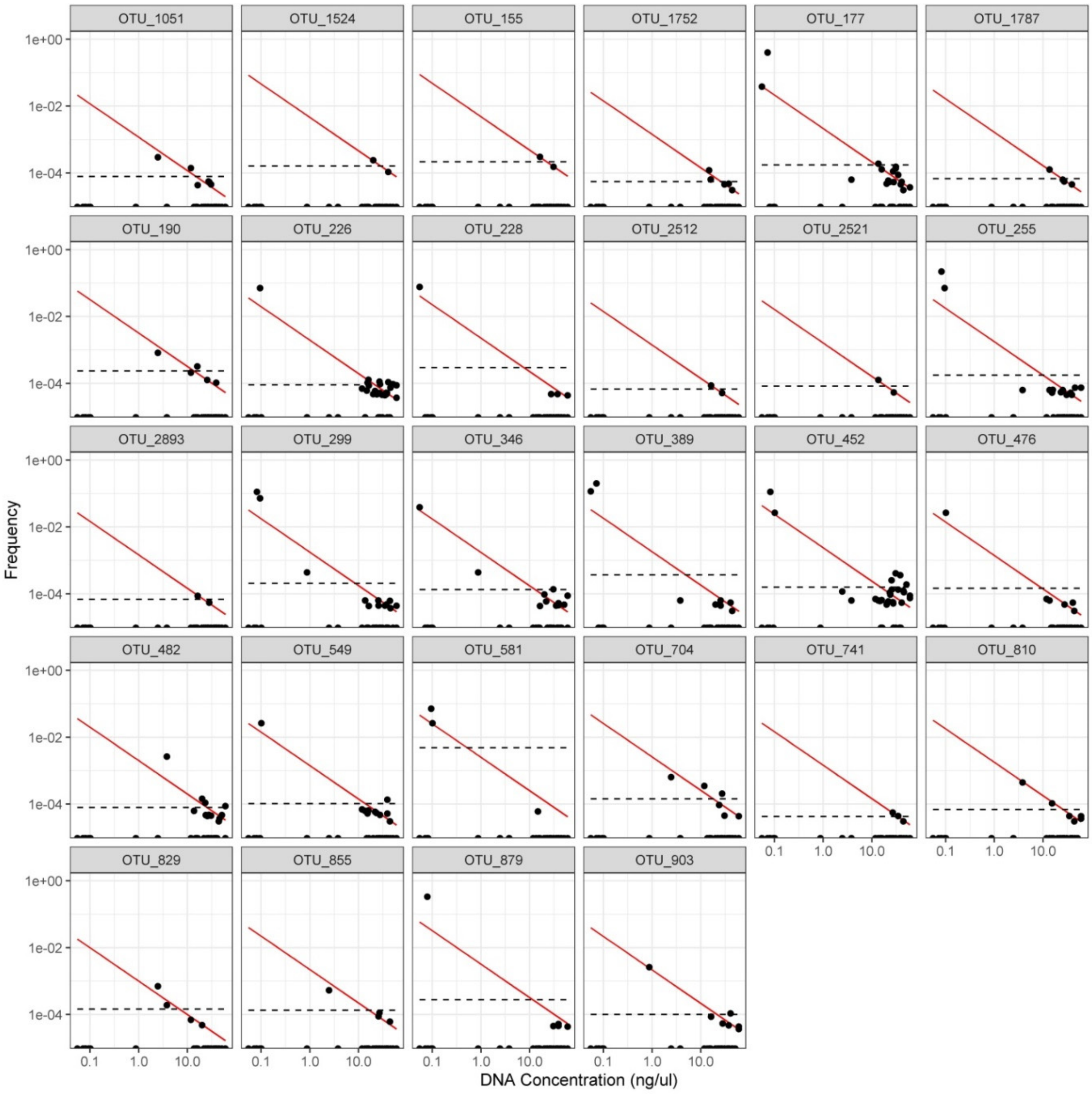

Figure S2. Contaminant OTUs identified in stool samples using decontam R-package. The frequencies of OTUs were inspected as a function of DNA concentration measured by Qubit assay prior to MiSeq sequencing. An OTU is classified as contaminant or non-contaminant by comparing its associated score statistic P (decontam score) to the default classification threshold

of 0.1. The frequency of all contaminant OTUs showed negative correlation with the total DNA concentration (red line). The horizontal black dot line represents the expected frequency of non-contaminant OTUs.

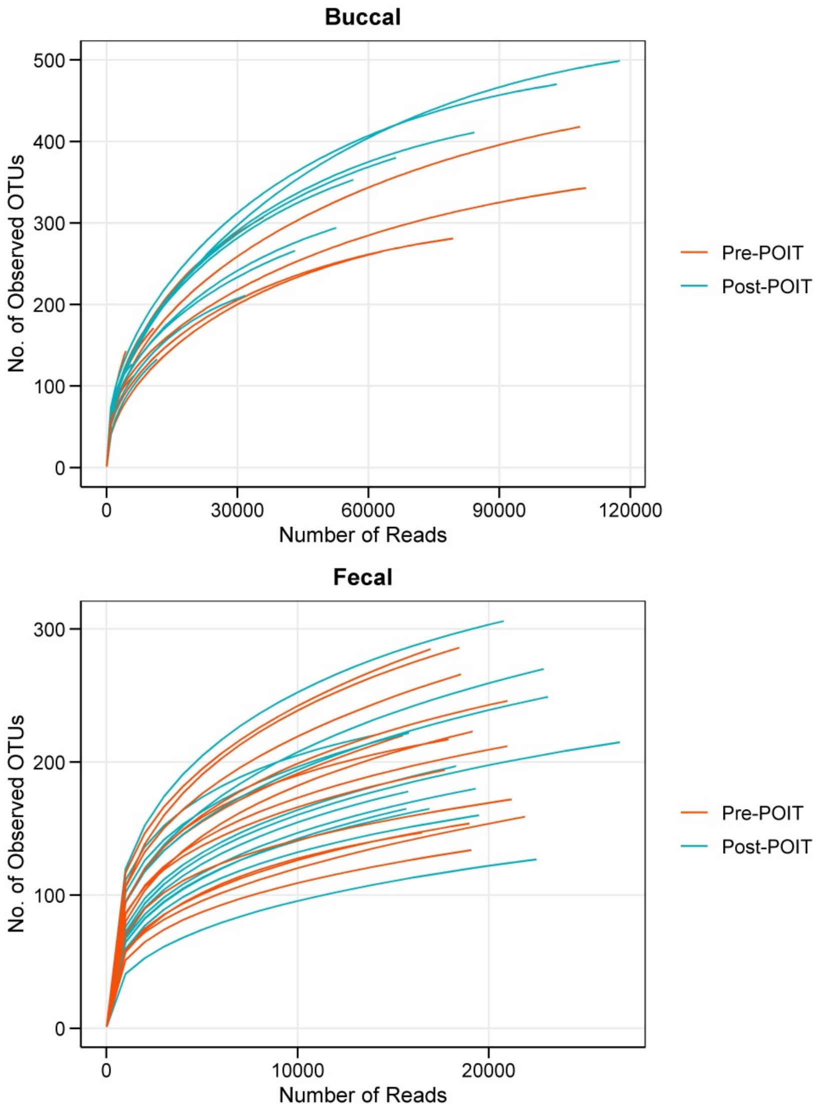

Figure S3. Rarefaction curves of observed OTUs vs read depths.

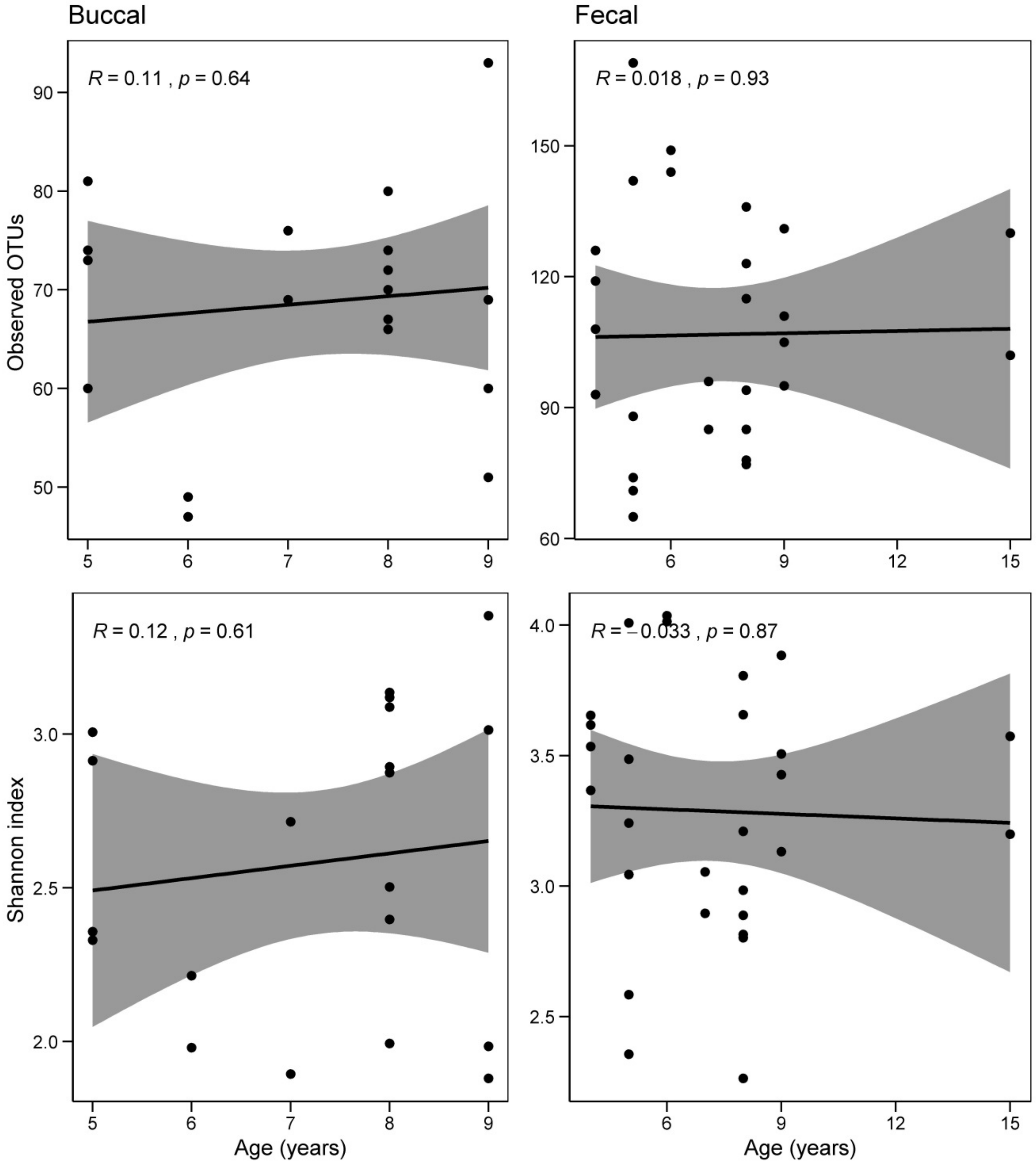

Figure S4. Correlations between alpha diversity and age of the participants. R=correlation coefficient.

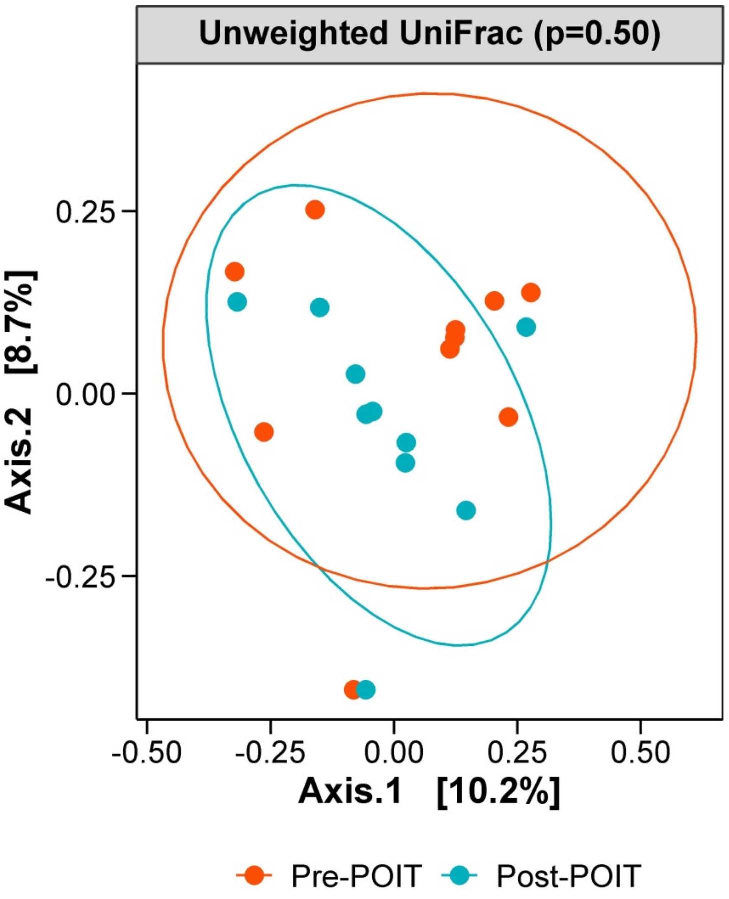

Figure S5. Unweighted UniFrac distance-based oral beta diversity in pre- and post-POIT.

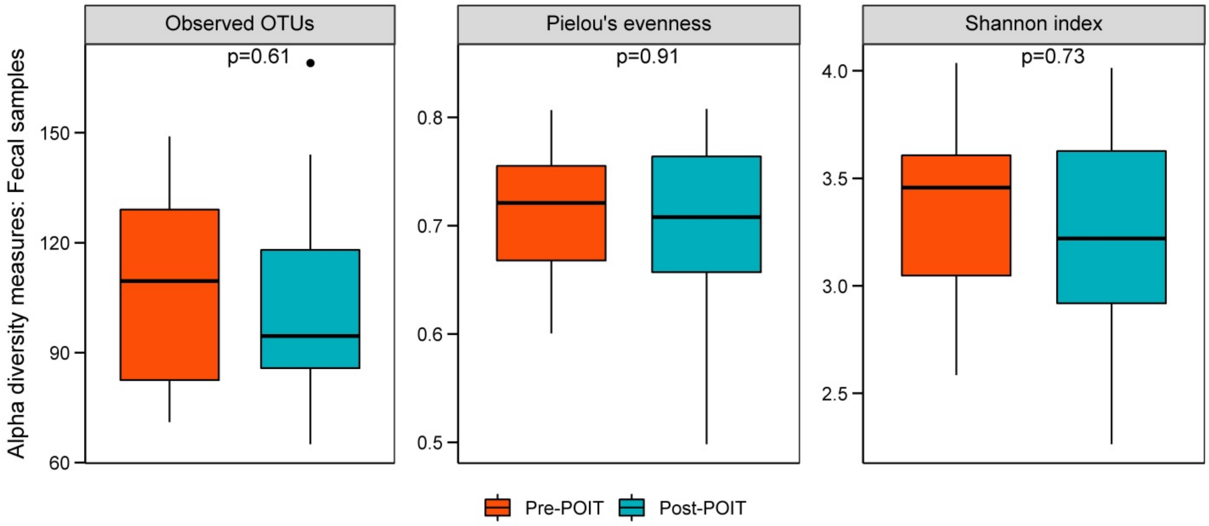

Figure S6. Alpha diversity of the fecal microbiome pre- and post-POIT.

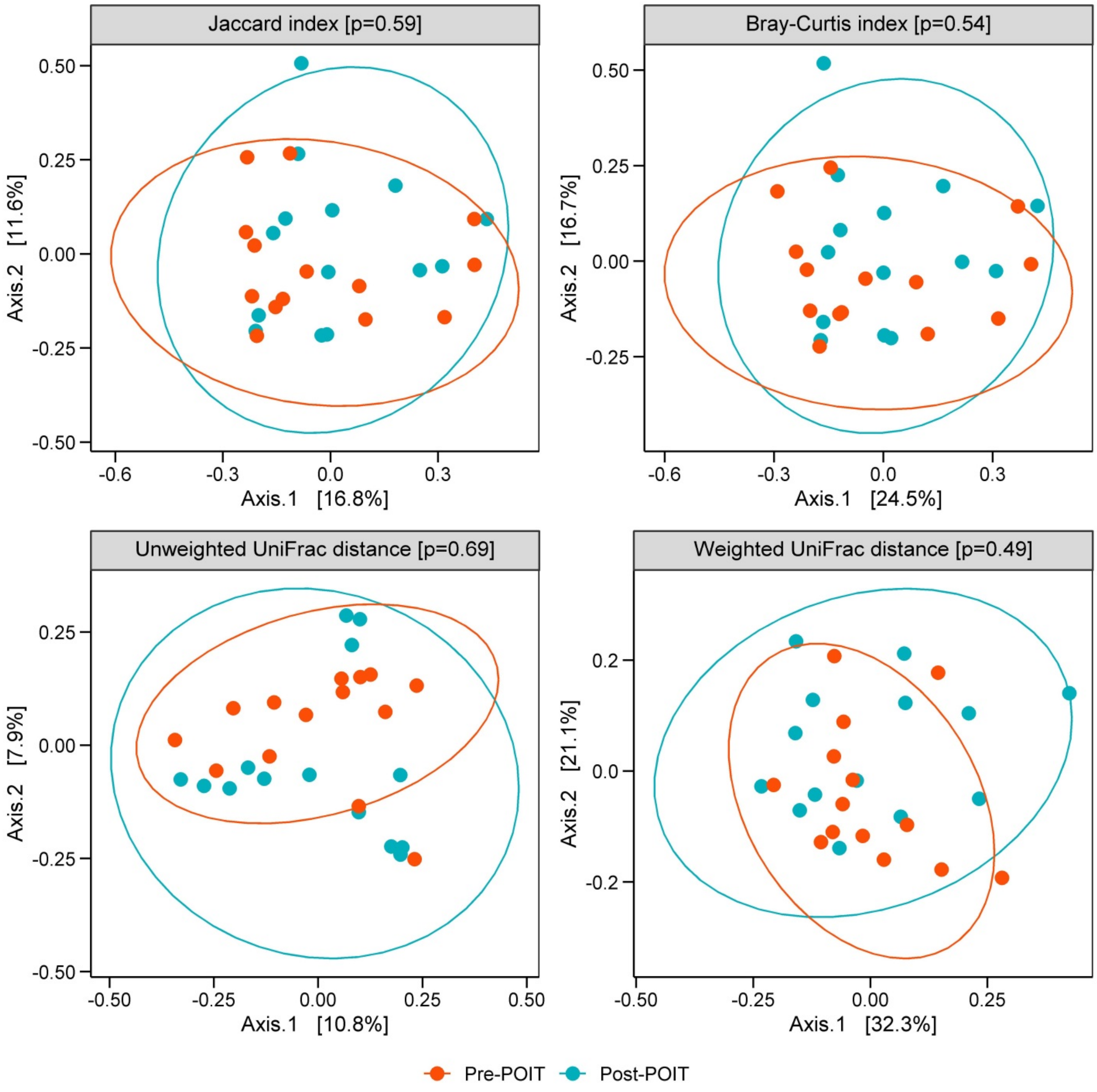

Figure S7. Beta diversity of the fecal microbiome pre- and post-POIT.

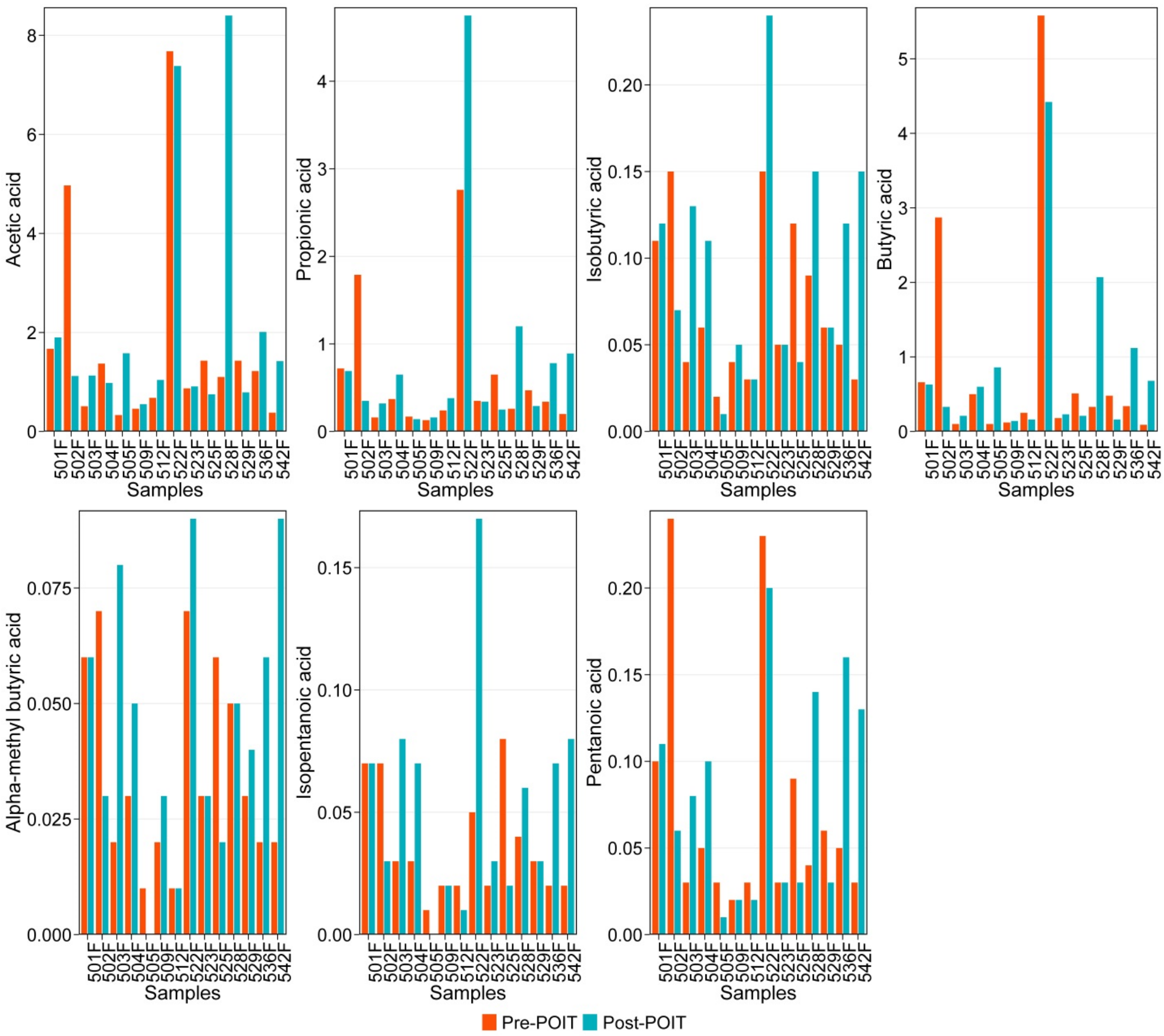

Figure S8. Fecal short chain fatty acids (SCFAs) levels in individual subjects before (pre) and after (post) POIT. SCFAs level was measured as µmole/g wet feces.

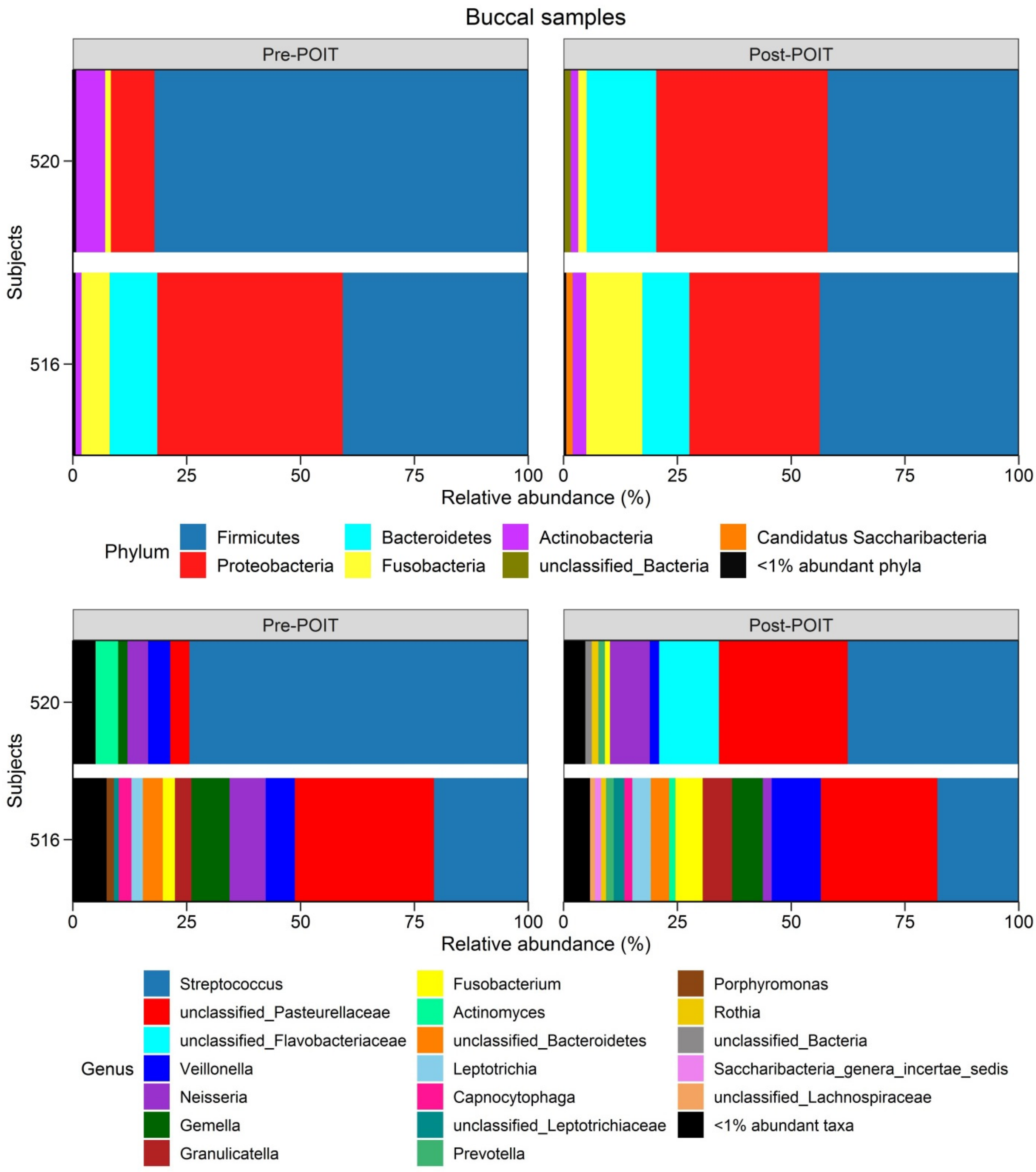

Figure S9. Oral bacterial composition in subjects taking probiotics during POIT.

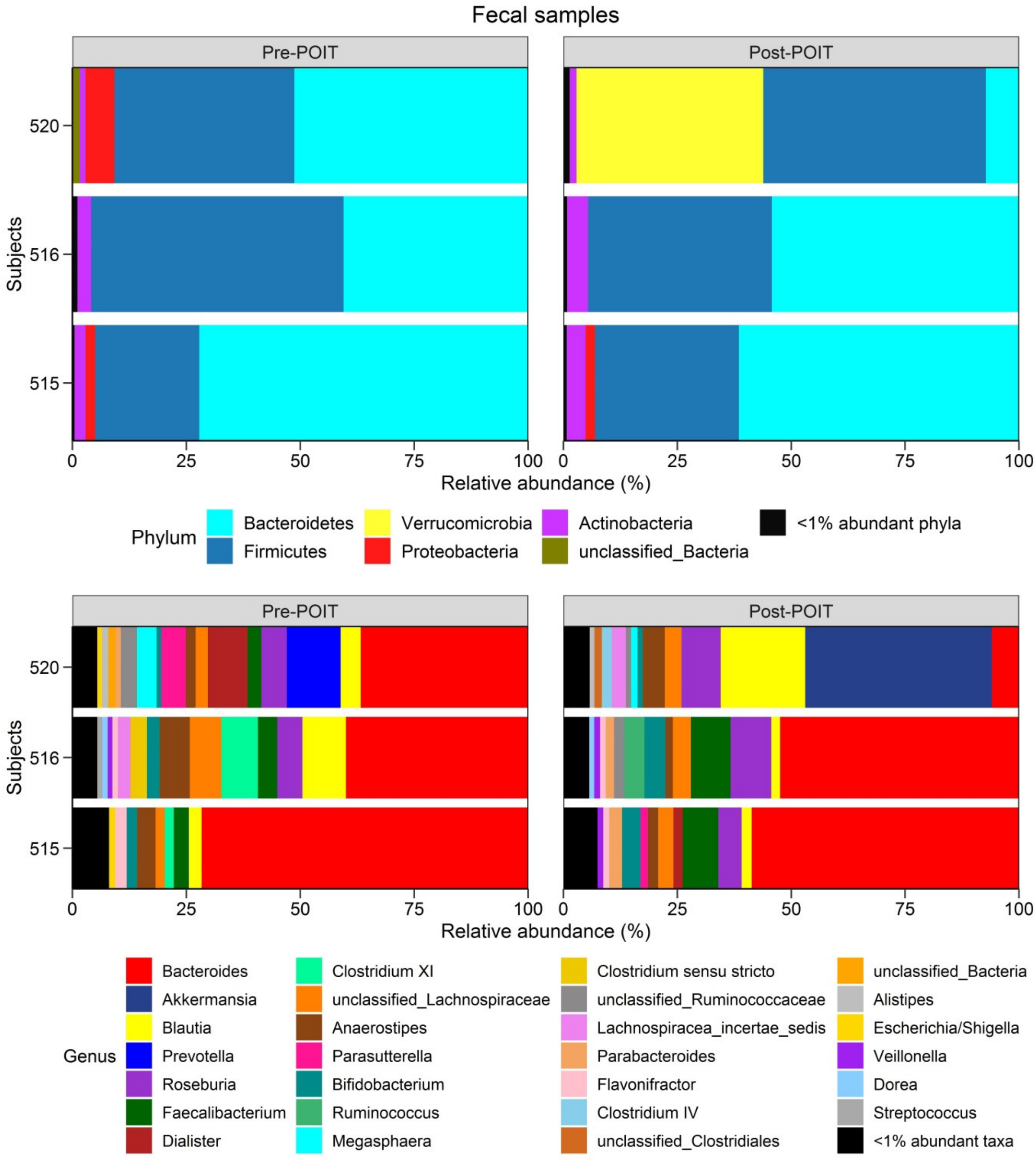

Figure S10. Fecal bacterial composition in subjects taking probiotics during POIT.

Table S1. Pre-POIT peanut-specific IgE and *Ara* h2 levels and POIT related-symptoms during each up-dosing level.

**Peanut-specific IgE** (kU/L)	***Ara* h 2** (kU/L)	**2 mg**	**5 mg**	**10 mg**	**25 mg**	**50 mg**	**100 mg**	**150 mg**	**200 mg**	**300 mg**
1.4	0.38	N (2)	AP (1)	None	N (2)	N, AP (6)	None	None	None	None
13.4	1.85	None	None	None	None	None	None	None	None	None
5.93	4.64	None	None	None	None	None	None	OI (4)	AP (1)	None
23.8	16.1	None	None	None	None	None	None	None	None	None
50.6	25.3	None	None	None	None	None	None	None	OI (1)	None
38.3	16.8	None	None	None	None	None	None	None	None	None
100	100	None	None	None	None	None	None	None	None	None
17.7	42.1	None	None	OI (1)	None	OI, H (1)	None	None	None	None
100	100	None	None	None	None	None	None	None	None	None
9.2	10.4	OI (1)	None	None	None	None	None	None	None	None
100	100	OI (7), V (1)	None	None	None	None	None	None	None	None
100	62.3	None	None	None	None	N, V (1)	OI, AP H, V (1)	None	N/A	None
100	76	None	None	None	None	None	OI (1)	None	N/A	None
75.9	53	None	H, AP (1)	AP (1)	AP (1)	AP, H (1)	None	None	N/A	None
11.5	16.3	None	OI (1)	None	None	None	OI (7)	OI (7)	None	None
15.7	16.2	None	None	None	None	None	P (1)	None	None	None
78.6	35.4	V (1)	None	V (1)	None	None	None	None	N/A	None

Number of occasions the symptoms occurred is mentioned in parenthesis. N=nausea, OI=oral itching, AP= abdominal pain, V=vomiting, H= hives, P=pruritus

Table S2: Cohort characteristics and demographics of individual participants.

Subject ID	Age (Y)	Sex	AD	Asthma	AR	Multiple FA	Delivery mode	Feeding	Anti-biotics	Pro-biotics	Birth place	Pets	Pets type	# Siblings, birth order & FA history
501	8	M	Yes	No	Yes	No	C	F	No	No	Urban	Yes	Dog	1_older
502	9	M	No	No	Yes	Yes	C	B	No	No	Urban	No	No	2 _older
503	5	M	Yes	No	No	Yes	C	B	No	No	Urban	Yes	Dog	1_younger brother_with PA
504	9	M	Yes	Yes	Yes	No	V	B	No	No	Urban	No	No	1_younger, 1older _egg allergy
505	5	M	Yes	No	Yes	Yes	V	B	No	No	Urban	No	No	2_older
509	6	M	No	No	Yes	Yes	C	B	No	No	Urban	Yes	rabbit	2_older
512	5	M	Yes	Yes	Yes	Yes	C	F	Yes	No	Urban	Yes	cats & dogs	1_older
515	4	M	Yes	No	No	Yes	C	F	No	Yes	NA	NA	NA	1_twin_with PA
516	4	F	Yes	No	No	No	C	F	No	Yes	NA	NA	NA	1_twin_with PA
520	9	F	Yes	Yes	Yes	Yes	V	B	Yes	Yes	Urban	No	No	2_younger sisters
522	7	M	Yes	No	Yes	Yes	V	B	Yes	No	Urban	Yes	Dog	2_younger
523	8	M	No	Yes	Yes	Yes	C	B	No	No	Urban	No	No	1older_with food allergies
525	4	F	Yes	Yes	No	No	C	B	Yes	No	Urban	No	No	1_younger
528	8	M	Yes	Yes	Yes	Yes	C	F	Yes	No	Urban	Yes	Dog	1_younger brother_with PA
529	4	M	Yes	Yes	Yes	Yes	C	B	No	No	Urban	Yes	Dog	1_older_with PA
536	15	M	Yes	Yes	Yes	Yes	V	B	No	No	Urban	Yes	Dog	1_younger
542	8	M	No	No	No	No	C	B	No	No	Urban	Yes	Dog	1_older

Abbreviations: F=female, M=male, AD=Atopic dermatitis, AR=Allergic rhinitis, FA=Food allergy, V=Vaginal, C=Cesarean-section, V=Vaginal, B=Breastfed, F=Formula.

Table S3: Effects of various factors on the oral and gut microbiome composition, as measured by PERMANOVA test of Bray-Curtis dissimilarity index.

**Variable**	**Oral microbiome** (n=20) Bray-Curtis			**Gut microbiome** (n=28) Bray-Curtis		
	F-value	R^2	p-value	F-value	R^2	p-value
Delivery mode	0.81	0.04	0.57	1.51	0.05	0.11
Feeding pattern	0.42	0.02	0.94	1.24	0.05	0.23
Pets in household	0.82	0.04	0.60	1.20	0.04	0.25
Antibiotics use[#]	0.29	0.02	0.99	0.62	0.02	0.86
Atopic dermatitis	0.81	0.04	0.59	1.00	0.04	0.42
Allergic rhinitis	1.98	0.10	0.07	1.65	0.06	0.08
Asthma	0.73	0.04	0.69	1.57	0.06	0.10
Multiple food allergies	0.96	0.05	0.43	1.10	0.04	0.33
POIT treatment	2.14	0.11	0.04*	0.87	0.03	0.54

[#]Antibiotics use during POIT. *Statistically significant. R^2=effect size.

Table S4. Fecal SCFAs concentrations in participants taking probiotics during POIT.

SCFAs (umol/g)	515		516		520	
	**Pre**	**Post**	**Pre**	**Post**	**Pre**	**Post**
Acetic acid	1.14	1.98	1.30	1.07	4.60	2.47
Propionic acid	0.52	1.05	0.52	0.33	2.33	1.02
Isobutyric acid	0.04	0.16	0.07	0.05	0.37	0.10
Butyric acid	0.30	0.97	0.21	0.31	3.53	1.06
Alpha-methyl butyric acid	0.02	0.08	0.03	0.02	0.20	0.04
Isopentanoic acid	0.02	0.10	0.04	0.03	0.25	0.05
Pentanoic acid	0.01	0.03	0.01	0.002	0.85	0.18

Pre=pre-POIT, Post=post-POIT. Participants 515, 516 and 520 were taking probiotics during POIT.

Text S1: Detailed protocol for measurement of fecal short chain fatty acids.

Materials and methods

Chemicals, Reagents, and Supplies

Optima LC/MS-grade acetonitrile (ACN), formic acid (FA), methanol (MeOH), and water were obtained from Thermo Fisher Scientific (Waltham, MA, USA). Authentic SCFA reference materials (acetic acid, propionic acid, butyric acid, isobutyric acid, pentanoic acid, isopentanoic acid, and 2-methybutyric acid) were obtained from Millipore-Sigma (Burlington, MA, USA). Reagents used in the derivatization of the SCFAs include aniline and [$^{13}C_6$]-aniline (for carbon-13 labeled IS preparations) and were obtained from Millipore-Sigma, and 1-(3-dimethylaminopropyl)-3-ethylcarbodiimide hydrochloride (EDAC), 2-mercaptoethanol, and succinic acid were obtained from Thermo Fisher Scientific. Polypropylene microfuge (1.5 mL) and centrifuge tubes (15 mL and 50 mL) were obtained from Genesee Scientific (San Diego, CA, USA). LC separations were performed using a 5.0-micron Viva BiPh Biphenyl (300Å, 100 x 1 mm) analytical column coupled to a 5.0-micron Viva Biphenyl (10 x 2.1 mm) guard cartridge from Restek (Bellefonte, PA, USA).

LC-MS/MS Equipment and Software

The LC-MS/MS system used for this study included a Shimadzu (Kyoto, Japan) Nexera X2 MP UHPLC system coupled to a Sciex (Washington, DC, USA) 6500 QTRAP hybrid triple-quadrupole/linear ion trap mass spectrometer. Instrument control and data acquisition was performed using Sciex Analyst® (Version 1.6.2). Peak integration, weighted (1/x) linear regression analysis of calibration curves, and unknown sample analysis was performed using Sciex MultiQuant™ 3.0.1 (Version 3.0.6256.0).

Analyte Stock Solution Preparations

An individual stock solution for each SCFA compound was prepared at a concentration of 100 mM each, and at a final volume of 1 mL in a solution of 1:1 ACN: water (see Table S5).

Table S5. Molar concentration and the volume dilutions to prepare 100 mM stock solutions for each SCFA compound measured.

SCFA reference material	Concentration (M)	Volume needed (µL)
acetic acid	17.4	5.7
propionic acid	13.3	7.5
butyric acid	10.8	9.3
isobutyric acid	10.7	9.3
pentanoic acid	9.1	11
isopentanoic acid	9	11.1
2-methylbutyric acid	9	11.1

Derivatization and Quenching Solution Preparations

For derivatization reaction procedures, stock solutions of aniline and [$^{13}C_6$]-aniline were each prepared at a concentration of 100 mM in a solution of 1:1 ACN: water. EDAC was prepared at a concentration of 100 mM in water. For reaction quenching procedures, individual stock solutions of 2-mercaptoethanol and succinic acid were each prepared in water at 100 mM, and 250 mM, respectively. The EDAC and 2-mercaptoethanol solutions were prepared fresh before each use.

Derivatized Analyte and IS Stock Solution Preparations

The derivatization reactions used in the preparation of the analyte (N-Phenyl-SCFA derivatives) and corresponding IS (N-([$^{13}C_6$]-Phenyl)-SCFA derivatives) was reported previously [1]. Briefly, the preparation of each individual derivatized SCFA analyte and IS stock solution was initiated by mixing a 10 µL volume of the 100 mM SCFA stock, a 100 µL volume of the 100 mM aniline solution (analyte preparation) or 100 mM [$^{13}C_6$]-aniline solution (IS preparation), and a 50 µL volume of the 100 mM EDAC stock solution. The mixture was vortex-mixed for 20 sec, and incubated at 4°C for 2h. After completion of the derivatization reaction, the solution was quenched by the addition of 50 µL volumes of a 100 mM 2-mercaptoethanol solution and a 250 mM succinic acid solution.

Working IS Solution Preparations

The Working IS-A (WIS-A) Solution was prepared at a concentration of 2.5 µM for each of the N-[$^{13}C_6$]-Phenyl-SCFA derivatives by adding a 13 µL volume of each IS Stock Solution into a 19.909 mL volume of an 80:20 water: acetonitrile solution. The WIS-A Solution was vortex-mixed for 15 sec prior to use, and was stored at -20°C while not in use. *Note: The WIS-A Solution is used as the diluent for all derivatized unknown samples prior to analysis.*

The Working IS-B (WIS-B) Solution was prepared at a concentration of 2.25 µM for each of the N-[$^{13}C_6$]-Phenyl-SCFA derivatives by mixing a 9 mL volume of the WIS-A Solution and a 1 mL volume of the 80:20 water: acetonitrile solution in vessel. The WIS-B Solution was vortex-mixed for 15 sec prior to use, and was stored at -20°C while not in use. *Note: The WIS-B solution is used for Combined Intermediate Solution and Calibration Standard preparations.*

Combined Intermediate Solution Preparation

A Combined Intermediate Solution was prepared by diluting a 26 µL volume of each of the derivatized analyte stock solutions into an 818 µL volume of Working IS-B Solution (2.25 µM of each N-[$^{13}C_6$]-Phenyl-SCFA derivative), yielding a final SCFA concentration of 100 µM. The Combined Intermediate Solution was vortex-mixed for 15 sec prior to use, and was stored at -20°C while not in use. *Note: The Combined Intermediate Solution is used for Calibration Standard preparation.*

Calibration Standard Preparations

The WIS-B solution was used as the diluent for the preparation of the Calibration Standards according to the serial dilution scheme outlined in Table S6.

Table S6. Serial dilution scheme for the N-phenyl-SCFA derivatized Calibration Standards using the WIS-B solution as the diluent.

Calibration Std	Concentration (nM)	Source Solution	Volume of Source Solution (µL)	Volume of Working IS-B Solution (µL)
F	10,000	Combined Intermediate Solution	100	900
E	2500	F	100	300
D	625	E	100	300
C	156	D	100	300
B	39.0	C	100	300
A	9.77	B	100	300

Study Sample Extraction and Derivatization Procedures
The SCFA content of the study samples was performed by adding a 1.25 mL volume of extraction solvent (1:1 ACN: water) to a 125 mg mass of wet stool sample, and the samples solvated samples were vortex-mixed at a high rotational speed until completely homogenized. The samples were then centrifuged at 18,000*g* for 10 mins. A 500 µL volume of clarified sample supernatant was transferred to a fresh sample vial and frozen at -80°C until the samples were derivatized. The derivatization reaction for the study samples was initiated by mixing a 10 µL volume of clarified sample supernatant, a 100 µL volume of the 100 mM aniline solution, and a 50 µL volume of the 100 mM EDAC stock solution. The mixture was vortex-mixed for 20 sec, and incubated at 4°C for 2h. After completion of the derivatization reaction, the solution was quenched by the addition of 50 µL volumes of a 100 mM 2-mercaptoethanol solution and a 250 mM succinic acid solution. After derivatization, a 10 µL volume of the derivatized sample was diluted in a 90 µL volume of the WIS-A Solution, and the sample was vortex-mixed for 15 sec. The entire volume of sample was transferred to autosampler injection vials, and a 10 µL volume of samples was injected on the LC-MS/MS system. The final SCFA concentrations were normalized by the mass of stool processed for each sample, and the results were reported in nM/mg stool.

Liquid Chromatography-Tandem Mass Spectrometry Methods
A reversed-phase chromatographic system was developed using a 5-micron Viva BiPh Biphenyl (300Å, 100 x 1 mm) analytical column coupled to a 5-micron Viva Biphenyl (10 x 2.1 mm) guard cartridge. Mobile phase A (MPA), mobile phase B (MPB), and needlewash solutions were water + 0.1% FA, acetonitrile + 0.1% FA, and 1:1 MeOH: water, respectively. The chromatographic gradient program included a column temperature of 50°C, an autosampler tray chilling temperature of 12°C, a mobile phase flowrate of 0.20 mL/min, and a gradient elution program specified as follows: 0-2.0 min, 5% MPB; 2.0-9.0 min, 5-50% MPB; 9.0-9.1 min, 50-5% MPB; 9.1-12.0 min, 5% MPB; with an overall cycle time of 12.4 min per acquisition, and an operation mobile phase backpressure of ~1750 psi at initial conditions. The MS divert valve was enabled and specified to divert the chromatographic eluate according to the following program: 0-2.0 min, divert to waste (Position B); 2.0-12.0 min, divert to MS source (Position A); 12.0 min, divert to waste (Position B). The retention times for each of the N-phenyl-SCFA derivatives are as follows: N-phenyl acetamide, 3.4 min; N-phenyl propanamide, 5.1 min; N-phenyl isobutanamide, 6.3 min; N-phenyl butanamide, 6.7 min; N-phenyl 2-methylbutanamide, 7.6 min; N-phenyl isopentanamide, 7.8 min; N-phenyl pentanamide, 8.2 min.

The TurboIonSpray® electrospray ionization (ESI) probe was installed in the Turbo V™ ion source and operated with the following source conditions: ionization mode polarity: positive; curtain gas (CUR): 20; TurboIonSpray™ voltage (IS): +5,000 V; source temperature (TEM): 200 °C; Ion Source Gas 1 (GS1; nebulization gas): 25 psi; Ion Source Gas 2 (GS2; heater gas): 25 psi. MS/MS operational parameters include the following: collisionally activated dissociation (CAD) gas pressure: "High"; Q1 and Q3 resolution setting: unit. The mass spectrometric instrument parameters are specified in Table S7 below.

Table S7. Mass spectrometric parameters for each of the N-Phenyl-SCFA derivative and N-[$^{13}C_6$]-Phenyl-SCFA derivatives. Abbreviations include mass-filtering quadrupole (Q1 and Q3), collision energy (CE), declustering potential (DP), entrance potential (EP), and cell-exit potential (CXP).

Q1 (m/z)	Q3 (m/z)	Time (mS)	ID	CE (eV)	DP (V)	EP (V)	CXP (V)
136.1	94	20	N-phenyl acetamide 136>94	+22	+60	+9	+15
136.1	77	20	N-phenyl acetamide 136>77	+40	+60	+9	+15
142.1	100	20	$^{13}C_6$-N-phenyl acetamide 136>100	+22	+60	+10	+15
142.1	84	20	$^{13}C_6$-N-phenyl acetamide 136>84	+51	+60	+10	+15
150.1	94	20	N-phenyl propanamide 150>94	+22	+70	+9	+15
150.1	77	20	N-phenyl propanamide 150>77	+42	+70	+9	+15

156.1	100	20	$^{13}C_6$-N-phenyl propanamide 156>100	+26	+60	+10	+15
156.1	82.9	20	$^{13}C_6$-N-phenyl propanamide 156>82.9	+45	+60	+10	+15
164.1	94	20	N-phenyl butanamide 164>94	+23	+75	+9	+15
164.1	77	20	N-phenyl butanamide 164>77	+45	+75	+9	+15
170.1	100	20	$^{13}C_6$-N-phenyl butanamide 170>100	+25	+60	+10	+15
170.1	82.9	20	$^{13}C_6$-N-phenyl butanamide 170>82.9	+49	+60	+10	+15
164.1	94	20	N-phenyl isobutanamide 164>94	+24	+72	+9	+15
164.1	77	20	N-phenyl isobutanamide 164>77	+46	+72	+9	+15
170.1	100	20	$^{13}C_6$-N-phenyl isobutanamide 170>100	+24	+72	+9	+15
170.1	82.9	20	$^{13}C_6$-N-phenyl isobutanamide 170>82.9	+46	+72	+9	+15
178.1	94	20	N-phenyl pentanamide 178>94	+23	+80	+9	+15
178.1	77	20	N-phenyl pentanamide 178>77	+48	+80	+9	+15
184.1	100	20	$^{13}C_6$-N-phenyl pentanamide 184>100	+23	+80	+9	+15
184.1	82.9	20	$^{13}C_6$-N-phenyl pentanamide 184>82.9	+48	+80	+9	+15
178.1	94	20	N-phenyl isopentanamide 178>94	+22	+80	+9	+15
178.1	77	20	N-phenyl isopentanamide 178>77	+48	+80	+9	+15
184.1	100	20	$^{13}C_6$-N-phenyl isopentanamide 184>100	+22	+80	+9	+15
184.1	82.9	20	$^{13}C_6$-N-phenyl isopentanamide 184>82.9	+48	+80	+9	+15
178.1	94	20	N-phenyl 2-methylbutanamide 178>94	+23	+80	+9	+15
178.1	77	20	N-phenyl 2-methylbutanamide 178>77	+49	+80	+9	+15
184.1	100	20	$^{13}C_6$-N-phenyl 2-methylbutanamide 184>100	+23	+80	+9	+15
184.1	82.9	20	$^{13}C_6$-N-phenyl 2-methylbutanamide 184>82.9	+49	+80	+9	+15

Reference

1. Chan, J. C., Kioh, D. Y., Yap, G. C., Lee, B. W., Chan, E. C., (2017) A novel LCMSMS method for quantitative measurement of short-chain fatty acids in human stool derivatized with 12C- and 13C-labelled aniline. *J. Pharm. Biomed. Anal. 138*, 43-53.
